# Development of a Screening Method for Fluoroquinolones in Meat Samples Using Molecularly Imprinted Carbon Dots

**DOI:** 10.3390/bios13110972

**Published:** 2023-11-07

**Authors:** Ahmed Faried Abdel Hakiem, Idoia Urriza-Arsuaga, Javier L. Urraca

**Affiliations:** 1 Pharmaceutical Analytical Chemistry Department, Faculty of Pharmacy, South Valley University, Qena 83523, Egypt; 2 Department of Analytical Chemistry, Faculty of Chemistry, Universidad Complutense de Madrid, 28040 Madrid, Spain; 3 Independent Researcher, 28007 Madrid, Spain; idoia.urriza@educa.madrid.org

**Keywords:** fluoroquinolones, molecularly imprinted polymers, carbon dots, nano-composites, fluorescence quenching, meat samples

## Abstract

An accurate and simple screening method has been developed for the determination of fluoroquinolone antibiotics. Carbon dots were synthesized by simple hydrothermal treatment as highly fluorescent nano-sensors. They were subsequently used in the synthesis of organic-based molecularly imprinted polymers to develop fluorescence-based polymeric composites using enoxacin as a representative dummy template molecule of fluoroquinolones. The method was optimized concerning the pH of the medium and composite concentration. The normalized fluorescence intensity showed efficient quenching under optimized conditions upon successive addition of the template, with an excellent correlation coefficient. The proposed method was applied to eight other fluoroquinolones, exhibiting, in all cases, good correlation coefficients (0.65–0.992) within the same linearity range (0.03–2.60 mg mL^−1^). Excellent detection and quantification limits were been obtained for the target analytes down to 0.062 and 0.186 mg L^−1^, respectively. All studied analytes showed no interference with enrofloxacin, the most commonly used veterinary fluoroquinolone, with a percentage of cross-reactivity varying from 89.00 to 540.00%. This method was applied successfully for the determination of enrofloxacin in three different types of meat samples: beef, pork, and chicken, with good recoveries varying from 70 to 100% at three levels. This new procedure is an easy analytical method that can be useful as a screening method for monitoring the environmental hazard of fluoroquinolones in quality control laboratories.

## 1. Introduction

Fluoroquinolones (FQs) are one of the most important classes of antibiotics, with excellent activity against pathogenic bacteria [1]. The antibiotic effectiveness of fluoroquinolones has contributed to their widespread use in food animals, such as chickens and cows. Fluoroquinolones are absorbed well after oral administration and distributed in animal tissues. The presence of these antibiotics in food induces pathogen resistance to clinical drugs [2,3,4] and has side effects on the central nervous systems of younger children [5], causing serious problems with human health. As a consequence, consumers and authorities are increasingly becoming concerned about the presence of residual antibiotics in the food supply due to their negative impact on human health. To ensure that consumers are not exposed to quinolone residues at potentially harmful concentrations, the European Union (EU) established maximum residue limits (MRLs) for such compounds present in edible tissues [6]. There are several methods for the determination of quinolones, typically based on high-performance liquid chromatography (HPLC) with fluorescent (FLD) or mass detectors (MS) and the enzyme-linked immunosorbent assay (ELISA). These methods have been widely used for the determination of quinolones in various matrices, such as food, water, and biological fluids. They are sensitive, specific, and reliable, making them valuable tools in the diagnosis and treatment of bacterial infections. Nevertheless, these methods are usually expensive or time-consuming.

New methods based on the use of molecularly imprinted polymers (MIPs) have become a good alternative to previous ones. MIPs are highly selective sorbents that have been used as specific molecular recognition materials for a variety of environmental and pharmaceutical analytes [7,8,9,10].

The most versatile approach to the synthesis of molecularly imprinted sorbents is based on non-covalent interactions between the template and the functional monomers. By the addition of a cross-linker and in the presence of heat or UV light, the polymer is formed. Finally, once the template molecule is extracted, a three-dimensional network is created with cavities that are complementary, in terms of size, geometry, and functional group orientation, to the template molecule. The material obtained is then ready to be directly implemented into the recognition of target molecules in sample extracts. Therefore, molecularly imprinted polymers (MIPs) can be considered as synthetic alternatives to immunosorbents, due to their high affinity and selectivity for several important target analytes. They often exhibit striking similarities to antibodies, primarily with regard to cross-reactivity and binding constants when compared with the corresponding immobilized antibodies. Focusing on fluoroquinolones, MIPs have been studied widely in the analysis of different samples, such as water [11], urine [12,13], foodstuff [14], and tissue samples [15].

Currently, the challenge in developing MIP sensors is not only to improve the selectivity and sensitivity of the manufactured materials, but also to make the imprinting process completely green [16,17]. For MIP synthesis, large amounts of reagents and organic solvents are needed. The same is true for the extraction, purification and cleaning processes. Moreover, MIP particles have poor degradability and tend to accumulate in ecosystems. Therefore, the negative effects of MIPs are related to both the fabrication process of MIP materials and waste production after MIP use. Great efforts must be made to find alternative methods to minimize the negative impact of MIP technology. This greenification framework includes proposals such as the use of renewable and innocuous starting materials (templates, functional monomers, and porogens), the use of smaller amounts of solvents, and the treatment of chemical residue waste.

Carbon dots (CDs) are tiny, carbon-based nanoparticles with unique optical and electronic properties [18]. These nanoparticles are a good alternative to fluorescent dyes and heavy-metal-based quantum dots due to their lower toxicity and their resistance to photobleaching [19]. They are typically smaller than 10 nm and can be synthesized using a variety of methods, including laser ablation, chemical oxidation, and hydrothermal synthesis. The use of renewable raw materials (plants) or renewable refined compounds (citric acid or amino acids) makes CD synthesis green. Carbon dots have a wide range of potential applications due to their unique properties. They are highly luminescent, typically in the ultraviolet and visible (UV-Vis) light range. They have been applied successfully in various applications, including bioimaging, sensing, and optoelectronics. CDs also have a high surface area and can be functionalized with various molecules, making them useful for drug delivery and other biomedical applications. Moreover, they are biocompatible, which means that they do not harm living organisms. CDs have also been used previously in the successful determination of FQs, using an inorganic polymer and carbon nanotubes [20].

**Figure 1:**
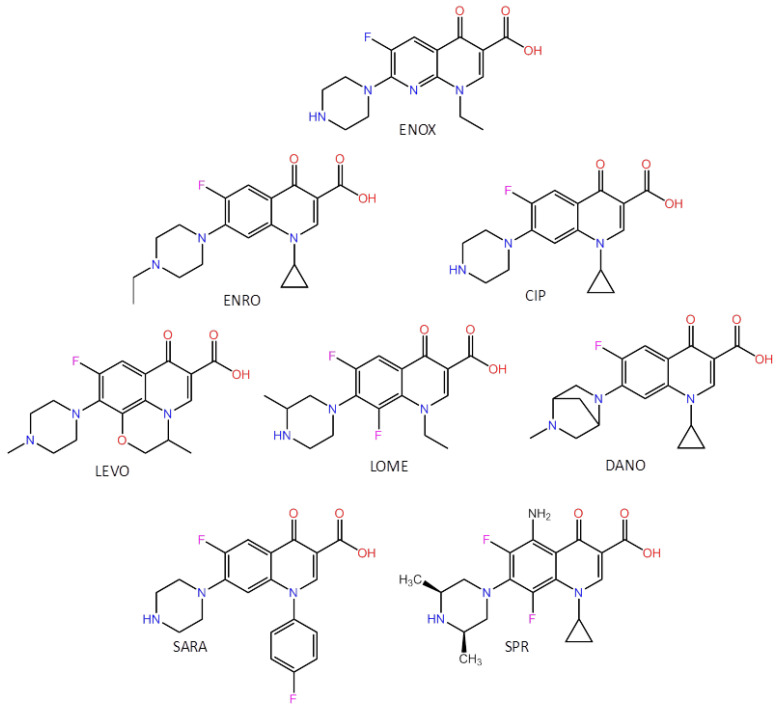
Chemical structures of the template molecule (ENOX) and the target FQs.

In this paper, we report the development of a novel ultrasensitive fluorescent probe (CDs@MIP) that combines the advantages of MIPs and CDs to specifically recognize and quantify FQs by simply encapsulating CDs in MIPs using a very simple synthesis and measurement procedure. CDs emitting were first synthesized as fluorophores from citric acid and urea by a hydrothermal green method. Then, molecularly imprinted polymers embedded with carbon dots were prepared by mixing CDs, enoxacin as a template, methacrylic acid and trifluoromethacrylic acid as functional monomers, and ethylene glycol dimethacrylate as a cross-linker. The new optical sensor exhibited high sensitivity and selectivity toward several FQ antibiotics (see Figure 1), being useful as a precise, simple, and rapid screening method for FQs in meat samples.

## 2. Materials and Methods

### 2.1. Chemicals

The next antibiotics, including the template molecule (Enoxacin, ENOX), ciprofloxacin (CIP), levofloxacin (LEVO), lomefloxacin (LOME), sparfloxacin (SPR), phosphoric acid, sodium phosphate, disodium phosphate, and trisodium phosphate, were obtained from Sigma-Aldrich (St. Louis, MO, USA). The initiator N,N′-azo-bis-(2,4-dimethyl)valeronitrile) (ABDV) was supplied by Wako (Neuss, Germany). Enrofloxacin (ENRO) was supplied by Fluka (Buchs, Switzerland). Sarafloxacin hydrochloride (SARA) was a gift from Fort Dodge Veterinaria (Girona, Spain). Danofloxacin (DANO) was obtained from Riedel-de-Haën (Seelze, Germany).

HPLC-grade solvents, including acetonitrile (AcN) and methanol (MeOH), were provided by SDS (Peypin, France). Water was purified using a Milli-Q system from Millipore (Bedford, MA, USA). Methacrylic acid (MAA), 2-(trifluoromethyl)acrylic acid (TFMAA), and ethylene glycol dimethacrylate (EDMA) monomers were purified as required using an inhibitor-remover from Aldrich (Milwaukee, WI, USA) immediately before use. Trifluoroacetic acid (TFA) (HPLC-grade, 99%) was obtained from Fluka (Buchs, Switzerland).

### 2.2. Apparatus

To establish the different values of pH (3.0–11.0) using several phosphate buffer solutions, an ORION 710A pH/ ISE meter (Beverly, MA, USA) was used. Luminescence measurements were carried out using a Fluoromax 2 spectrofluorometer (Horiba Jobin-Yvon, France). A Nicolet 6700 FTIR Advanced Gold Spectrometer supported with OMNIC 8 software (Thermo Electron Scientific Instruments Corp., Madison, WI, USA) was for data processing. To prepare the KBr PNQDs, sample discs were used, along with a 15 mm glass mortar and pestle and a hydraulic press with a Perkin Elmer die press and a Thermo-Scientific (Fischer, Waltham, MO, USA) Qwik handi-press instrument. For the TEM images, a high-resolution electron microscope (HRTEM) was used (JEM-100-CXII plus JEOL microscope working at 120 kV).

### 2.3. Synthesis of Carbon Dots (CDs)

The CDs were synthesized as in previous work [21], but with some modifications to make the process simpler. Accurately weighed amounts of citric acid (4.2 g) and urea (3.6 g) were dissolved in 40 mL of milli-Q water and transferred to a 100 mL Teflon-lined stainless-steel autoclave for hydrothermal treatment at 180 °C for four hours. The obtained water-soluble colloidal solution was cooled to room temperature. The final suspension was used directly for further experiments.

### 2.4. Synthesis of the Molecularly Imprinted Polymers—Quantum Dots Composites

Twenty milligrams of the template molecule (ENOX) were mixed with 10 µL of MAA and 17 mg of TFMAA as functional monomers, and 226 µL of the cross-linker (EDMA) in a 24 mL glass vial. Then, the vial was sealed with a septum and the mixture was purged with N_2_ for 15 min. After another 15 min under stirring, 3 mL of NQDs colloidal solution dispersed in 2 mL of DMSO and 15 mL of AcN was added to the vial. The resulting mixture was purged again with N_2_ for 10 min and it was further stirred for another 15 min. Finally, 6 mg of the initiator (ABVD) was added and the system was purged with N_2_ for another 10 min. The mixture was stirred overnight with gentle heating at 40 °C. The collected composites were washed four times (20 min each time at 8000 rpm) with a mixture of methanol–trifluoracetic acid at 90:10 (*v*/*v*). Non-imprinted composite polymer (CDs–NIP) was prepared using the same procedure, but in the absence of the template molecule.

The obtained composites were diluted with MilliQ water to obtain 20 mL aqueous suspensions. Then, a volume of 1.0 mL of MIP or NIP was transferred to previously weighed 2 mL vials and dried at 100 °C in an oven. After being cooled to room temperature, the 2 mL vials were weighed again to obtain the exact concentrations of both suspensions. Both MIP and NIP solutions were further diluted with water to obtain suspensions of 100 mg L^−1^.

The synthesized CDs, as well as the MIP composites, were characterized by FTIR spectroscopy and transmission electron microscopy (TEM). A few drops of the colloidal aqueous solutions of NQDs and the aqueous suspension of composite MIP were ground and mixed well separately with pure KBr crystals. The mixture, which was dried in an oven at 40 °C, was compressed into thin discs for FTIR measurements. TEM was used for further examination of the physical properties of the nano-sized colloidal CDs as well as the suspended MIP–CDs composite particles.

### 2.5. Spectrofluorimetric Measurements

The excitation wavelength (λ_ex_) and emission wavelength (λ_em_) were set at 346 and 393 nm, respectively. The signal was normalized to obtain the calibration curves, subtracting the residual fluorescence of every solution, over the solution containing the CDs@MIPs according to the next equation: S = (B−X)/(B_0_−X), where S is the normalized signal, B is the fluorescence signal of the analyte in the presence of CDs@MIPs, X is the residual signal of every solution in the absence of the CDs@MIPs, and B_0_ is the fluorescent signal of the CDs@MIPs in the absence of analyte. For the optimization of the assay, MIP and NIP suspensions were prepared in the concentration range of 0.45 to 50 mg L^−1^ at different pH values from 3 to 11 using 50 mM phosphate buffer. The validation study was based on the construction of calibration graphs for ENOX and the whole investigated FQs, at the same linearity range. The detection limit (LOD) was calculated as the FQ concentration for which the fluorescent signal was reduced by 10%. For cross-selectivity studies, the calibration curves for all the analytes in water were obtained in the concentration range of 0.33–2.60 µg mL^−1^.

### 2.6. Preparation of Fortified Meat Samples

Into 24 mL glass vials, 1 g samples of beef, pork, and chicken meat (three samples of each) were transferred and diluted with 5 mL of phosphate buffer, pH = 7. The samples were sonicated for 15 min. The obtained aqueous suspensions were centrifuged at 8000 rpm for 15 min. The clear supernatants were then transferred using Pasteur pipettes into clean glass vials and were subjected to ultrafiltration through a 0.22 µM Millipore filter using a peristaltic pump. Then, 50 µL of the composite aqueous suspension (100 mg L^−1^) was added to a 5 mL clear glass vial. The resulting suspension was diluted up to 3.00 mL with the filtered clear supernatant of the filtered ENRO-fortified meat sample solution at the correct concentration level. All analyses were performed in triplicate.

## 3. Results and Discussion

### 3.1. Synthesis of the CDs and CDs@MIPs

**Figure 2:**
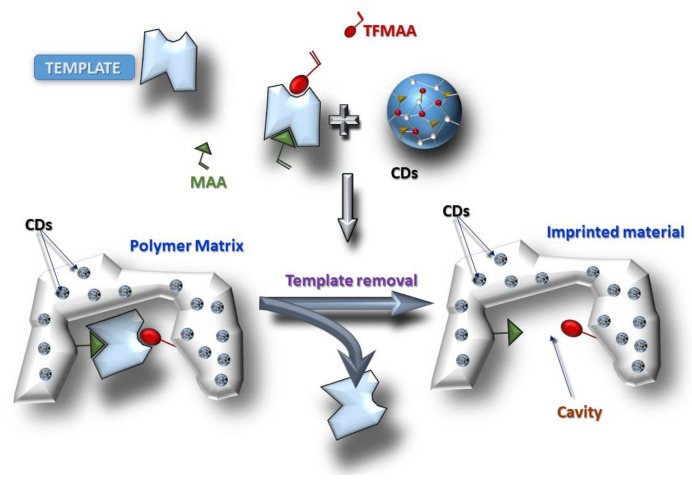
Scheme of the synthesis of the CDs@MIPs.

The synthesis of the CDs started with a condensation polymerization process between citric acid and urea, resulting in the formation of a polymer-like material. Subsequently, carbonization of the aforementioned material led to the creation of CDs. The synthesis of the CDs@MIPs was similar to that reported by Zhang et al., 2015 [21] (Figure 2), eliminating some purification steps. The polymers were obtained after the addition of the CDs to the prepolymerization mixture providing the fine core-shell particles. The synthesis template/functional monomer/cross-linker molar ratio of the MIP was set constant at 1:2:2:20. As described in previous work [22], the use of MAA/TFMAA as functional monomers for FQs achieved excellent imprinting using ENOX as the template molecule. The use of a mixture of DMSO/AcN as a porogen favored the electrostatic interactions or hydrogen bonding between the functional monomers and ENOX. The ratio between the volume of solvent used over the total amount of solvent plus monomer volume was above 0.99, greater than the ratio used in usual bulk polymers, which is around 0.57 [23]. This ratio was used to avoid the agglomeration of the polymer nanoparticles. In order to obtain homogeneous particles, the polymerization process was carried out under magnetic stirring.

### 3.2. Characterization of the CDs and CDs@MIPs

The analysis of the CDs FTIR spectrum (Figure S1 in Supplementary Materials) revealed a strong broad band at 3440 cm^−1^ corresponding to the stretching vibration of the hydroxyl moiety. The very weak band at 2950 cm^−1^ represents the stretching of both C–H and (C–C) ring vibrational modes. The weak band at 2920 cm^−1^ was attributed to the stretching vibration of the methyl moieties, while that at 2850 cm^−1^ corresponded to the combination of in-plane carbonyl moiety and (C–C) ring vibrational modes. The medium band at 1690 cm^−1^ and the medium-strong one at 1590 cm^−1^ were assigned to the C=O and C=C stretching vibrations, respectively. The medium-strong band at 1400 and the medium shoulder at 1340 were the bending vibration modes of the CH_2_ moiety. The weak vibrational band at 1003 cm^−1^ was considered the in-plane bending of the CCC mode, while the weak one at 835 cm^−1^ represented the bending mode of HCCH torsion. On the other hand, the FTIR spectrum of the composite was also studied. The medium-weak band at 2980 cm^−1^ was attributed to the stretching vibration of C–H aromatic bonds, while the other close medium-weak bands at 2960 and 2920 cm^−1^ corresponded to the combination of C–H and (C–C) ring vibrational modes. A combination of in-plane carbonyl moiety and (C–C) ring vibrational modes was represented by the weak band at 2850 cm^−1^. The very weak bands at 2350, 2230, and 2040 cm^−1^ were assigned to the nitrile moiety (C≡N). The carbonyl moiety appeared as a very strong sharp band at 1730 cm^−1^, while the alkene (C=C) corresponded to a medium vibrational band at 1630 cm^−1^. The bands at 1510 and 1450 cm^−1^ represented the stretching vibrational modes of the aromatic (C=C) bonds. The bands at 1390, 1260, and 1160 cm^−1^ were attributed to C–N stretching, while the medium shoulder at 1050 cm^−1^ referred to C–O stretching. The bending vibrational mode of the hydroxyl moiety was represented by two weak bands at 950 and 877 cm^−1^. Finally, the weak band at 750 cm^−1^ represented the bending vibration of C–H. Therefore, the confirmation of the polymerization process was achieved.

**Figure 3:**
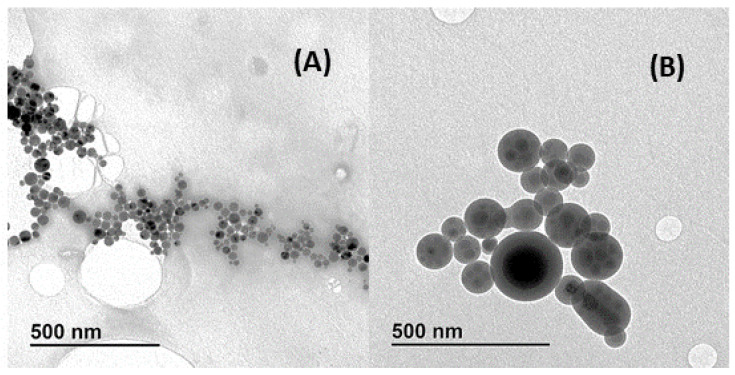
CD (**A**) and MIP@CD (**B**) nanoparticles.

CDs and MIP@CDs were also characterized by TEM images. Figure 3A shows the isolated CDs with a wide distribution of size particles (between 4 and 20 nm) and a slight agglomeration between them. This wide distribution of the CDs was due to the purification steps that were eliminated in comparison with Zhang et al.’s method [21], but the rendered material was still perfectly useful. Once the polymerization of the MIP/NIP took place, a moderated agglomeration of the CDs was produced. Then, multicore nanoparticles of the MIP@CDs were obtained with a distribution size in the range of 4–210 nm (Figure 3B). No agglomeration was observed for individual MIP@CDs particles.

Once the target analyte was bound to the composite material, the multicore particles of the MIP@CDs favored a greater variation in the CDs luminescence with respect to the original luminescence in the absence of the analyte. In the absorption spectra (not shown), two peaks were found around 250 and 350 nm. The first peak was related to the π-π* transfer of the aromatic ring, and the second peak was related to the n-π* transfer of the carbonyl group. As the maximum emission wavelength was obtained at an emission wavelength of 399 nm, this wavelength was considered optimal for all experiments.

### 3.3. Optimization of the Assay Conditions

**Figure 4:**
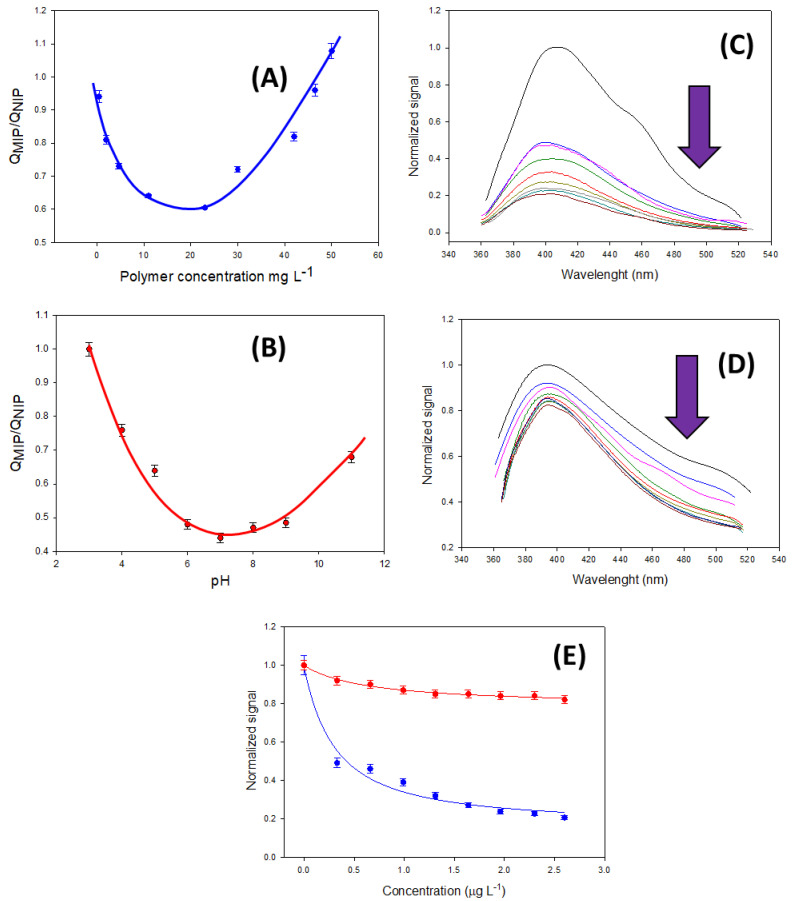
Quenching of the MIP over the quenching of the NIP as a function of the (**A**) polymer concentration (n = 3) and (**B**) pH (n = 3). (**C**,**D**) Variation in the normalized signal in the MIP and the NIP for different concentrations of ENRO (n = 3). From top to bottom: 0, 0.33, 0.66, 0.99, 1.31, 1.64, 1.96, 2.30, and 2.60 mg L^−1^. (**E**) Calibration curves of ENRO for the MIP (blue line) and NIP (red line) (n = 3).

To achieve the most sensitive conditions in the determination of FQs, certain parameters had to be optimized. As is the case in the immunological assays based on antibodies, the variation in the luminescence signal depends on the receptor concentration (antibodies or in this work the polymer). For this reason, the best MIP and NIP concentrations were first evaluated. With this purpose, for a fixed concentration of 1 mg L^−1^ of ENOX (pH = 5, 50 mM phosphate buffer), several concentrations of MIP and NIP were tested. The imprinting factor in this assay can be understood as the result of the quenching obtained in the MIP over the quenching obtained in the NIP. Figure 4 shows this variation for the ranges of concentrations. From this figure, it can be observed that the best conditions were obtained for a concentration of 23 mg L^−1^ (QMIP/QNIP = 0.605, n = 3, RSD 4.6%). As it was expected, a decrease in the polymer concentration increased the specific quenching in the presence of ENOX due to the specific number of binding sites also decreasing, as was the case for the B_0_ signal. This effect occurred from 450 mg L^−1^ (not plotted in the graph) up to 23 mg L^−1^. Nevertheless, for smaller concentrations of this value, this effect disappeared, probably due to the loss of the original signal B_0_, where the luminescence signal was conditioned by the sensitivity of the instrument.

On the other hand, the specific recognition of the polymer to ENOX is pH-dependent. This can be explained by taking into account the more potent acidic nature of the TFMAA monomer compared with MAA (TFMAA has a pKa of 3.1, whereas MAA has a pKa of 4.8), which favors the formation of hydrogen bonds with the piperazine moiety of the antimicrobial compound. When neutral pHs were studied (Figure 4B) the total deprotonation of functional monomers was achieved, while the piperazine moiety in ENOX remained protonated. For higher pH values, the deprotonation of the amine group also took place, decreasing the imprinting effect. Therefore, a pH value of 7 was selected for further experiments. Figure 4C,D shows the calibration curves for ENRO under the optimized conditions for a concentration range of 0–2.60 mg L^−1^. Finally, the fluorescence emission spectra of MIP@CDs and NIP@CDs at the excitation wavelength of 345 nm are shown in Figure 4C,D for the range concentration of 0–2.60 mg L^−1^ of ENRO.

### 3.4. Assay Analytical Characterization

The experimental data obtained for the surrogate molecule (ENOX) were fitted to a non-linear polynomial fitting through an external calibration. The data exhibited an excellent correlation coefficient (R^2^) of 0.9920 in the range studied range (0.03–2.60 mg L^−1^) with limits of detection and quantification of 0.07 and 0.22 mg L^−1^, respectively. Upon application of the developed composite to the other analytes at the same range, successful correlation coefficients ranging from 0.65 up to 0.991 were obtained with detection and quantification limits of 0.06 and 0.18 mg L^−1^, respectively. The limit of detection (LOD) and the limit of quantification (LOQ) were calculated as the analyte concentration for inhibiting the signal of the MIP@CDs by 10 and 50% respectively. All the experiments were conducted at room temperature and the laboratory was kept at 25 °C. Due to the very low concentration of the used material and the difficulty of recovering this very small amount of material in every measurement for this concentration level, every measurement was carried out with fresh material (µg L^−1^).

Experiments were conducted to determine cross-reactivity by examining the competitive curves of various FQs that could potentially be found in food samples under optimized conditions. Cross-reactivity was calculated by comparing the AC50 value for ZON with the AC50 value for the interfering compounds (as shown in Table 1). The obtained percentage cross-reactivity values ranged from 85.00 up to 512.00%, confirming the suitability of the developed method for the assessment of the environmental hazard of the other seven investigated FQs. The results of accuracy and precision in terms of intraday reproducibility (n = 3) are also included in Table 1, with a RSD (%I) of <5.1 for all the antibiotics. In addition, the material showed high stability over time (>6 months) without the loss of the properties, which is suitable for the routine analysis of these antibiotics.

### 3.5. Application to Meat Samples

The optimized method was applied for the determination of ENRO, which could be found in meat, in three different meat samples: beef, pork, and chicken. The samples were treated as mentioned in the experimental section. Three different concentrations of ENRO (50, 75, and 100 ng g^−1^, n = 3) were spiked to the obtained ultrapure-fortified meat samples. The same samples were also analyzed by HPLC-FLD. Thus, the CDs@MIP assay was validated from the studied values. No significant differences were observed using these two techniques. Nevertheless, a relevant decrease in the theoretical recoveries was observed in pork samples. This can be also observed in the determination by HPLC-FLD and it can be attributed to any matrix effect due to the interaction between the ENRO molecule and other co-extracted substances present in the sample. The findings indicated that the developed method, in conjunction with the extraction procedure, was appropriate for ENRO determination in this kind of sample (Table 2).

A comparison of this method with some reported works on FQs detection is also summarized in Table S1. HPLC-MS/MS methods are highly sensitive, but require expensive and sophisticated equipment, tedious sample preparation steps, and use large amounts of environmentally unfriendly organics solvents [24]. Jian et al. [25] reported an improved HPLC-MS/MS method by using a green extraction solvent and faster sample preparation. HPLC-FLD techniques [26,27,28] are less sensitive compared with the former ones. However, these methods are faster and simpler, and try to use lower volumes of organic solvents. Optical sensors [29,30,31] are easier and faster detection devices compared with LC-MS and HPLC methods, but are less sensitive. The new reported method combines good sensitivity with the advantages of optical sensors. Yuphintharakun et al. [20] developed a method similar to that in this work using an inorganic-based polymer. The analytical parameters rendered in that work were better when compared with this one. Nevertheless. a much more complex synthesis was required (nanowires are also involved in that process). Also, the use of the CDs@MIP presented in this work solves the well-known problems associated with inorganic-based polymers, such as: stability, compatibility with organic solvents, decreasing pore size with time, etc. On the other hand, the present method had, in some cases, a lower limit of detection compared with other reported methods, even when these reported methods have not been applied to the analysis of meat samples. The greater complexity of these samples versus other kinds of samples, such as urine and water, must also be considered in the comparison. Furthermore, this kind of method can be implemented in the determination of a wider group of FQs than the methods reported up to now.

## 4. Conclusions

This research has afforded an easy, fast, and straightforward screening method for the selective and sensitive determination of several FQs in meat samples. This method has overcome the disadvantages of the current existing expensive or time-consuming methods. The high sensitivity of the method was achieved by the combination of highly fluorescent nano-quantum dots with the selectivity provided by the MIP material. These formulated nano-composites have shown good correlation coefficients and a linearity range with excellent sensitivity down to 0.03 mg L^−1^. Upon application of the proposed method to different investigated analytes, including ENRO, CIPRO, DANO, SARA, SPAR, LEMO, and LEVO, excellent correlation coefficients were obtained at the same linearity range. The results obtained support the suitability of the method for the determination of the structurally related fluoroquinolones. This method was successfully applied to the determination of ENRO in different meat samples (beef, pork, and chicken) at three different ENRO concentrations. Excellent recoveries were obtained, from 70 to 100%. The developed method could provide an efficient simple analytical scheme for the assessment of fluoroquinolones in general, and especially ENRO, in control laboratories.

## Figures and Tables

**Table 1:** Detection limits, quantification limits, 50% inhibition, and cross-reactivity of the FQs studied.

FQs	LOD (µg mL^−1^)	LOQ (µg mL^−1^)	AC50 (µg mL^−1^)	Reproducibility (%) (n = 3)	Cross-Reactivity (%)
Enoxacin	0.070	0.211	0.345	4.3	100.0
Enrofloxacin	0.067	0.221	0.331	3.7	95.9
Ciprofloxacin	0.062	0.186	0.371	3.8	107.5
Levofloxacin	0.327	0.982	1.788	5.1	512.2
Lomefloxacin	0.072	0.220	0.420	3.6	121.7
Danofloxacin	0.065	0.195	0.294	4.3	85.2
Sarafloxacin	0.188	0.564	0.814	4.2	235.9
Sparfloxacin	0.432	1.296	0.785	4.0	227.5

**Table 2:** Recoveries studied with CDs@MIP assay and by HPLC for three different concentrations of ENRO (50, 75, and 100 ng g^−1^) in beef, pork, and chicken meat samples.

Sample	Spiked Level (ng g^−1^) (n = 3)	CDs@MIP Assay	HPLC-FLD
Beef	50	50 ± 4	51 ± 2
	75	70 ± 5	74 ± 2
	100	83 ± 8	90 ± 4
Pork	50	42 ± 4	41 ± 5
	75	52 ± 6	60 ± 6
	100	75 ± 9	80 ± 8
Chicken	50	48 ± 3	50 ± 3
	75	75 ± 5	76 ± 2
	100	85 ± 7	92 ± 6

## Data Availability

The data presented in this study are available on request from the corresponding authors.

## Supplementary Materials

The following supporting information can be downloaded at: https://www.mdpi.com/article/10.3390/bios13110972/s1, Figure S1: FTIR spectra of the MMC nanoparticles before (red line) and after (black line) MIP coating; Table S1: Comparison of various methods for the determination of FQs. References [20,25,26,27,28,29,30,31,32,33,34,35,36,37,38,39,40,41,42,43] are citated in the supplementary materials.
