# Supplementary material for: Development of a Screening Method for Fluoroquinolones in Meat Samples Using Molecularly Imprinted Carbon Dots
Source: Biosensors (Basel). 2023 Nov 7;13(11):972. doi: 10.3390/bios13110972 (PMC10669409; doi:10.3390/bios13110972)
Supplement: Supplementary file 1 [file biosensors-13-00972-s001.zip › biosensors-2665546-supplementary.pdf]

Supplementary Material

Development of a Screening Method for Fluoroquinolones in Meat Samples Using Molecularly Imprinted Carbon Dots

Ahmed Faried Abdel Hakiem [1,2,*], Idoia Urriza-Arsuaga [3] and Javier L. Urraca [2,*]

[1] Pharmaceutical Analytical Chemistry Department, Faculty of Pharmacy, South Valley University, Qena 83523, Egypt
[2] Department of Analytical Chemistry, Faculty of Chemistry, Universidad Complutense de Madrid, 28040 Madrid, Spain
[3] Independent Researcher, 28007 Madrid, Spain; idoia.urriza@educa.madrid.org
* Correspondence: ahmed@fariedharm.kfs.edu.eg (A.F.A.H.); jurracar@ucm.es (J.L.U.)

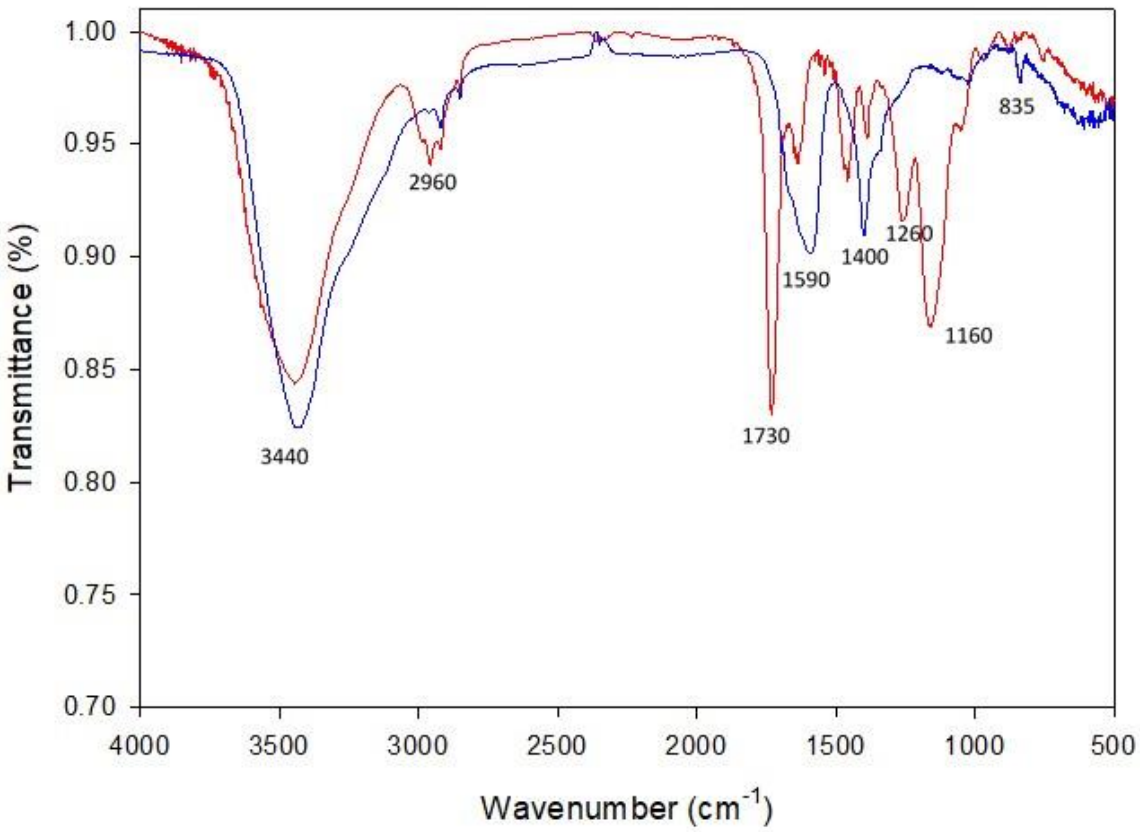

Figure S1. FTIR spectra of the MMC nanoparticles before (red line) and after (black line) MIP coating.

Table S1. Comparison of various methods for the determination of FQs.

Method	Analyte	LOD (µg mL^{-1})	LOQ (µg mL^{-1})	Sample	References
Poplar flower carbon dots (Pf-CDs)	ENRO	0.027	-	Milk and tap water	[32]
Mn-doped ZnS quantum dots (QDs)	CIPRO	0.15	0.50	Serum and urine samples	[33]
Nitrogen-doped fluorescent carbon dots (N-CDs)	ENRO	0.16	0.53	Tap water and river water	[34]
Yellow fluorescent carbon dots (Y-CDs)	NOR<br>CIPRO	0.0054<br>0.012	-<br>-	Tablets and milk samples	[35]
Sulfur-doped carbon dots (S-CDs)	NOR<br>CIPRO	0.0015<br>0.0022	-<br>-	Bovine raw milk	[36]
Carbon dots (CDs)	LEVO	0.00072	-	Water and milk samples	[37]
Uniform N- and S-co-doped carbon dots (NSCDs)	LEVO	0.0051	-	Water samples	[38]
Carbon dots (CDs)	CIPRO	0.0002	-	Eel<br>Grass carp<br>Freshwater shrimp	[39]
Lanthanide metal–organic frameworks	CIPRO<br>NOR<br>ENRO<br>ENOX	0.0056<br>0.35<br>0.0057<br>0.018	-	Tap water and river water	[40]
Micellization and metal complexation	LOME	0.0038	0.0114	Tablet and eye drop formulations	[41]
Quantum dots (QDs) incorporated into MIP	CIPRO	0.00007	-	Chicken muscle and milk	[42]
Own fluorescence of FQs	ENOX<br>ENRO<br>CIPRO<br>LEVO<br>LOME<br>DANO<br>SARA	0.2<br>0.3<br>0.4<br>0.2<br>0.5<br>0.2<br>0.3	0.6<br>1.3<br>1.3<br>0.6<br>2<br>0.6<br>1	Medicinal preparations	[43]
Nanocomposite optosensor	CIPRO	0.000066	0.00022	Chicken and milk samples	[20]
µSPE–UHPLC–MS/MS	NOR	Milk 0.012 µg kg^{-1}<br>Muscle/liver/kidney 0.012 µg kg^{-1}<br>Egg 0.024 µg kg^{-1}	0.04 µg kg^{-1}<br>0.04 µg kg^{-1}<br>0.08 µg kg^{-1}	Milk, animal muscle, liver, kidney, and egg	[25]
	CIPRO	Milk 0.024 µg kg^{-1}<br>Muscle/liver/kidney 0.060 µg kg^{-1}<br>Egg 0.060 µg kg^{-1}	0.08 µg kg^{-1}<br>0.20 µg kg^{-1}<br>0.20 µg kg^{-1}		
	LOME	Milk 0.016 µg kg^{-1}<br>Muscle/liver/kidney 0.024 µg kg^{-1}<br>Egg 0.012 µg kg^{-1}	0.06 µg kg^{-1}<br>0.08 µg kg^{-1}<br>0.04 µg kg^{-1}		
	ENRO	Milk 0.024 µg kg^{-1}<br>Muscle/liver/kidney 0.024 µg kg^{-1}<br>Egg 0.012 µg kg^{-1}	0.08 µg kg^{-1}<br>0.08 µg kg^{-1}<br>0.04 µg kg^{-1}		
Fluorescence detection	LOME	Milk: 0.00155<br>Beef: 0.00078	0.00468<br>0.00236	Milk and beef	[29]
	CIPRO	Milk: 0.00020<br>Beef: 0.00103	0.00061<br>0.00311		
MSPE–UV-Vis	CIPRO	0.04 nmol g^{-1}	0.15 nmol g^{-1}	Meat samples	[30]
Optical biosensor	ENRO<br>CIPRO	1 ppb	-	Meat and milk samples	[31]
SPE–HPLC–FLD	CIPRO<br>DANO<br>ENRO<br>SARA	0.2 mg kg^{-1}<br>0.04 mg kg^{-1}<br>0.1 mg kg^{-1}<br>0.2 mg kg^{-1}	0.5 mg kg^{-1}<br>0.1 mg kg^{-1}<br>0.4 mg kg^{-1}<br>0.6 mg kg^{-1}	Animal feed	[26]
SBSME–HPLC–FLD	CIPRO<br>LOME<br>ENRO<br>LEVO	0.1 ng g^{-1}	0.3 ng g^{-1}	Milk	[27]
MAE–SPE–UHPLC–FLD	CIPRO	6.0 ng g^{-1}	20.2 ng g^{-1}	Fish	[28]

HPLC–MS/MS	CIPRO	0.01	-	Human aqueous humor	[24]
Our method	ENOX<br>ENRO<br>CIPRO<br>LEVO<br>LOME<br>DANO<br>SARA	0.070<br>0.067<br>0.062<br>0.327<br>0.072<br>0.065<br>0.188	0.211<br>0.221<br>0.186<br>0.982<br>0.220<br>0.195<br>0.564	Beef, pork, and chicken samples	This work

SPE: Solid-phase extraction; UPLC: Ultra-high-performance liquid chromatography; MSPE: Magnetic solid-phase extraction; SBSME: Stir bar sorptive microextraction; MAE: Microwave-assisted extraction; MG-DSPE: Magnetic grapheme-based dispersive solid-phase extraction.
